# Using genetics to explore complement C5 as a druggable protein in periodontitis

**DOI:** 10.3389/fimmu.2024.1407431

**Published:** 2024-10-08

**Authors:** Zoheir Alayash, Sebastian-Edgar Baumeister, Birte Holtfreter, Thomas Kocher, Hansjörg Baurecht, Benjamin Ehmke, Stefan Lars Reckelkamm, Michael Nolde

**Affiliations:** ^1^ Institute of Health Services Research in Dentistry, University of Münster, Münster, Germany; ^2^ Department of Restorative Dentistry, Periodontology, Endodontology, and Preventive and Pediatric Dentistry, University Medicine Greifswald, Greifswald, Germany; ^3^ Department of Epidemiology and Preventive Medicine, University of Regensburg, Regensburg, Germany; ^4^ Clinic for Periodontology and Conservative Dentistry, University of Münster, Münster, Germany

**Keywords:** complement C5, immunomodulation, periodontitis, drug discovery, instrumental variable analysis

## Abstract

**Aim:**

An excessively activated or dysregulated complement system has been proven to be a vital contributor to the pathogenesis of periodontitis. It has been previously hypothesized that inhibiting the activity of complement component C5 by targeting the C5a receptor is a powerful candidate for treating periodontitis. Here, we apply the drug target instrumental variable (IV) approach to investigate the therapeutic effect of genetically proxied inhibition of C5 on periodontitis.

**Method:**

In our primary analysis, we used 26 independent ‘cis’ single nucleotide polymorphisms as IVs from the vicinity of the encoding locus of C5 that are associated with plasma C5 levels. In a secondary analysis, we assess the validity of our primary findings, exploring the involvement of alternative downstream biomarkers, interleukin 17 (IL-17), interleukin 1β (IL-1β), and tumor necrosis factor (TNF). Summary statistics of plasma levels (C5, IL-17, IL-1β, and TNF) were obtained from a genome-wide association study (GWAS) of 35,559 European descent individuals. We extracted association statistics from a GWAS of 17,353 clinical periodontitis cases and 28,210 European controls. Wald ratios were combined using inverse-variance weighted meta-analysis.

**Results:**

In our primary approach, inhibiting C5 reduced the risk of periodontitis (Odds ratio 0.89 per 1 standard deviation reduction in C5; 95% confidence Interval 0.80–0.98, *p* value=0.022). Our secondary analysis suggests an involvement of IL-17 within the potential causal pathway, but was inconclusive for other biomarkers.

**Conclusions:**

The findings from our study suggest that C5 inhibition may reduce the risk of periodontitis, prioritizing C5 inhibitors as a potential adjunctive therapeutic intervention in this disease.

## Introduction

Periodontitis is a chronic inflammatory condition caused by a complex intricate interplay between dysregulated host immunomodulation and polymicrobial dysbiotic communities that form on subgingival tooth sites (1). Susceptibility to periodontitis can be influenced by genetic factors that may predispose to excessive inflammation, as well as environmental factors (e.g., stress and diet), risk-related behavior (e.g., smoking) and diseases (e.g., diabetes mellitus and rheumatoid arthritis) that can detriment the host immune response (2–5). The unregulated inflammatory response can lead to the destruction of the teeth-supporting structure, and if left untreated, lead to tooth loss, impaired mastication and compromised aesthetics (6). Periodontitis affects around 50% of adults, with around 10% suffering from severe periodontitis (7). This disease impacts oral health and exhibits associations with other comorbidities, including cardiovascular diseases, diabetes mellitus, rheumatoid arthritis, and Alzheimer’s disease (4). The implications of periodontitis extend beyond oral health, emphasizing the importance of an effective treatment to prevent the onset or the progression of other diseases.

Periodontitis treatment is usually done by first improving the patient’s oral hygiene and controlling risk factors. Then, in the second step, professional removal of supra and subgingival biofilm and calculus is performed, with or without adjunctive treatments. Although there have emerged a list of adjunctive therapies over the years, only systemic antibiotics are being considered in advanced cases (8). Emerging evidence from novel drug candidates suggests a promising future for adjunctive therapeutic strategies in treating periodontitis. Among these candidates, complement component inhibitors stand out as noteworthy due to the extensive evidence implicating complement in the pathogenesis of periodontitis (9).

The complement cascade can be initiated by distinct mechanisms (classical, lectin, or alternative), which converge at complement component 3 (C3). The complement system plays a crucial role in innate host defense through three main pathways: lysis, inflammation, and opsonization/phagocytosis. During complement activation, the formation of the Membrane Attack Complex leads to the targeted lysis of cells by disrupting their membranes. Anaphylatoxins such as C3a and C5a, generated during this process, are potent proinflammatory molecules that bind to their respective receptors on immune cells, triggering G-protein coupled signaling. Their physiological functions include hallmark proinflammatory activities, such as increasing vascular permeability, inducing smooth muscle contraction, recruiting leukocytes, and enhancing white blood cell responses through chemotaxis, migration, and phagocytosis. Additionally, anaphylatoxins promote the production and release of other inflammatory mediators, like interleukins. The complement system also facilitates the tagging and clearance of pathogens via opsonization, where fragments like C3b bind to complement receptors on phagocytic cells, such as macrophages and neutrophils, promoting the engulfment and removal of foreign bodies (10).

C3 has been a focal point of investigation (11); however, little is known about the involvement of C5 in periodontal diseases. Given its placement in the complement cascade and the mechanistic similarities between C3 and C5, it is plausible to hypothesize that C5 may exert a comparable influence on periodontal inflammatory processes. For instance, C5a levels were more pronounced in patients with periodontitis in gingival crevicular fluids and saliva (12). In addition, the knockout of C5a receptor gene and the topical application of a C5a inhibitor (PMX-53) in mice models have demonstrated the ability to halt periodontal inflammation and inflammatory markers, mainly interleukin 17 (IL-17), interleukin 1β (IL-1β), interleukin 6 (IL-6), and tumor necrosis factor (TNF-α) (13, 14). We acknowledge that the immune inflammatory response in periodontitis is complex, involving both innate and acquired immunity and thus a wide variety of inflammatory biomarkers. The pathogenic roles of TNF, IL-1β, and IL-6 in causing destructive periodontal inflammation in both humans and animal models are particularly well documented (15, 16). Recent studies have also highlighted the pathogenic role of IL-17, which has been linked to periodontitis due to its elevated levels in diseased gingiva and gingival crevicular fluid (17, 18). Furthermore, a direct association between IL-17 and periodontal bone loss has been demonstrated in mice (19).

In this article, we employ an instrumental variable (IV) approach to determine whether there is a causal relationship between the inhibition of C5 and periodontitis risk and explore the relevance of certain inflammatory cytokines on this relationship. This study design uses single nucleotide polymorphisms (SNPs) as IVs to link modifiable risk factors to disease outcomes (20). Recently, this approach has been developed to investigate the relationship between a druggable protein, like C5, and disease risk, like periodontitis (21). Due to random mating, Mendel’s laws of segregation, and independent assortment, SNPs are distributed without dependence on traits that they do not directly affect. Thus, genetic variants can be treated analogously to random treatment assignment in a randomized controlled trial (22). It is worth noting that limited research has explored the relationship between C5 inhibition and periodontitis. Nevertheless, the availability of C5 genome-wide association studies (GWAS) and the periodontitis GWAS created the opportunity to study the potential therapeutic impact of the genetically proxied inhibition of C5 using cis-IVs on periodontitis risk. The genetic evidence from this study predicts the probability of success in future clinical trials and offers valuable insights into the relevance of inflammatory biomarkers in the C5-periodontitis axis. Hence, our study would enhance interest in investigating this relationship through preclinical and clinical studies.

## Method

### Study design

Our IV method is a drug target approach that utilizes genetic variants from the vicinity of a gene locus known to encode a druggable protein, like C5, to predict the potential therapeutic effect of C5 inhibition on periodontitis risk. To ensure the validity of the instruments and infer causality, three assumptions must be satisfied: 1) relevance; strong instruments-risk factor association, 2) independence; no common causes of instruments and the outcome, 3) exclusion restriction/no-horizontal pleiotropy; instruments solely impacting the outcome through the exposure (23). The causal inference derived from this study design minimizes the impact of bias from confounding and reverse causation and makes our study analogous to a randomized controlled trial (24). Genetic variants carried from parents to offspring are randomly allocated during conception; hence, they are independent of observed and unobserved confounders of the exposure-outcome association (25). Also, genetic associations are guarded against reverse causation since genetic variants remain fixed throughout one’s lifetime and are not altered by the disease process (22).

### Instrument selection

A way to inhibit the activity of C5a can be achieved by inhibiting its precursor, C5. We selected instruments to proxy the inhibition of C5a activity using cis-variants associated with plasma C5 levels [within 1Mb of the C5 gene-coding region chr9:120,932,987-121,075,195 (GRCh38/hg38)] at a stringent level of significance (*p* value< 1*10^-5^). We performed LD clumping r^2^< 0.1 to ensure SNPs were independent. SNPs can be inherited together if they are in proximity; thus we clump the SNPs that are associated with a trait so we do not use correlated IVs in our analysis. The use of correlated IVs would underestimate the variability of causal estimate. This procedure retained 26 independent SNPs with an F-statistic ≥10, reducing the risk of weak instrument bias (**Supplementary Table S1**).

In our secondary analysis, we explored the effect of C5 inhibition using an alternative way, assessing whether periodontal inflammation is linked to the downregulation of IL-17, IL-1β, and TNF via C5. Hence, we searched SNPs from the C5 gene-coding region associated with these downstream biomarkers’ plasma levels. It is important to note that previous research has focused on a list of important inflammatory markers that are associated with periodontal inflammation. However, whether these biomarkers are relevant to the C5-periodontitis axis is not well established. We noticed that none of the SNPs identified in the vicinity of the C5 gene was associated with any relevant biomarker at a stringent threshold (*p* value< 1*10^-5^). Since our study only considered cis-SNPs located in a single locus, there is no need to compensate for the inflation of type one error to the same extent as it is done in genome-wide association studies, and it is reasonable to set the *p* value at < 1×10^-3^. Performing clumping at r^2^< 0.1, two SNPs were associated with IL-17 and IL-1β, one SNP was associated with TNF, and none was associated with IL-6. All selected SNPs had F-statistics ≥10 (**Supplementary Tables S2**-**S4**).

For our causal estimates to be considered valid, several key assumptions shall be satisfied. The genetic variants used as proxies must be strong (the relevance assumption); the association of the genetic variants with periodontitis may not be confounded (the independence assumption); and the association of the genetic variants with the outcome is may not be explained by pathways independent of the druggable protein (the exclusion restriction) (26). The exclusion restriction/no-horizontal pleiotropy assumption cannot be verified in our study. In the general context of an IV approach, where IVs are chosen irrespective of their proximity to the encoding gene, a set of sensitivity analyses are usually employed to assess the validity of this assumption (23); however, in our cis- approach, the SNPs were selected based on biological plausibility. Genetic variants within the vicinity of the protein-encoding gene are less likely to exhibit horizontal pleiotropic effects compared to more distant variants (21).

### Periodontitis GWAS

The Gene-Lifestyle Interactions in Dental Endpoints consortium provided summary statistics for periodontitis. A total of 17,353 participants of European ancestry were classified as clinical periodontitis cases and 28,210 as controls. Periodontitis was defined by either the Centers for Disease Control and Prevention/American Academy of Periodontology classification or the Community Periodontal Index case definition (27).

### Drug target GWAS (C5, IL-17, IL-1β, IL-6 and TNF)

All variants were selected from the Gene- encoding loci of C5. In our primary analyses we used summary statistics on the association between the variant and C5. For our secondary analysis we selected variants from the C5 encoding loci as well but from GWAS of the respective proteins to obtain association statistics of the variants and IL-17, IL-1β, IL-6 and TNF. Variants were derived from a GWAS of plasma protein levels conducted in the Age, Gene/Environment Susceptibility cohort, a large-scale proteogenomic study (N= 35,559). Association estimates in this GWAS were derived from participants of European descent. Plasma protein levels were measured with the SomaScan version 4 assay (SomaLogic) (28).

### Statistical analysis

We estimated the effects for each variant using the Wald ratio and pooled these estimates using inverse variance weighted (IVW) meta-analysis. Additionally, we performed a leave-one-out analysis to assess if the causal effect substantially changes upon removing a single instrument (23). We reported the odds ratio (OR) and 95% confidence (CI) per standard deviation increment of circulating C5 levels as proxied by the IVs. All analyses were performed in R version 4.1.2 using the TwoSampleMR and MendelianRandomization packages.

## Results

All instruments used were highly associated with plasma levels of the respective protein, and none reported an F-statistic of less than 10. Second, we searched the IVs used in our analyses in Phenoscanner and confirmed that none is associated with any known confounders. In our primary analysis, C5 inhibition was linked to a reduced risk of periodontitis (OR 0.89 [95% CI 0.80, 0.98]). A protective effect was also observed in our secondary analysis, focusing on the downstream biomarker IL-17, where C5 inhibition was associated with a decreased risk of periodontitis (OR 0.24 [95% CI 0.20, 0.30]). However, we observed no conclusive effect estimates of C5 inhibition when considering other downstream biomarkers. The analysis showed an OR of 0.54 [95% CI 0.21, 1.35] for IL-1β and 0.40 [95% CI 0.05, 2.93] for TNF (**Table 1**). In a leave-one-out analysis, we demonstrated that single instruments neither changed the effect direction nor heavily influenced the overall estimate (**Supplementary Figure S1**).

**Table 1:** Mendelian Randomization estimates for effects of C5 inhibition on periodontitis using genetic variants as instruments.

	No. SNPs	OR	95% CI	*p* value
Primary analysis				
C5	26	0.89	(0.80; 0.98)	0.0229
Secondary analysis				
IL-17	2	0.24	(0.20; 0.30)	0.0000
IL-1β	2	0.54	(0.21; 1.35)	0.1873
TNF	1	0.40	(0.05; 2.93)	0.3652

IL-17, interleukin 17; IL-1 β, interleukin 1 β; tumor necrosis factor; CI, confidence interval; IVW, inverse-variance weighted analysis; OR, odds ration per one standard deviation decrease in C5.

## Discussion

The present study applied a genetic design to index a druggable protein and provides a cost-effective approach to prioritize drug targets and predict drug response in future clinical trials. In our analysis, involving 17,353 periodontitis cases and 28,210 controls, we applied a cis-IV approach to study potential effect of C5 inhibition on periodontitis risk. Our results support C5 as a viable candidate in preventing and treating periodontitis. We found supporting evidence for a potential involvement of IL-17 in the disease mechanism. Results were not conclusive using the other examined inflammatory biomarkers IL-1β, IL-6 and TNF.

Our findings showed a decreased risk of periodontitis due to genetically proxied inhibition of C5. This study represents a significant contribution to the field, in which there is currently only one comparable study, which is based on a mouse model. In this study, local administration of PMX-53, a C5a antagonist, effectively inhibited periodontal inflammation and reduced bone loss (13). Notably, considerable research has focused on complement C3 inhibition in periodontal diseases. The main studies are a Phase IIa clinical trial (11) and another recent study similar in design to our current study that explored the effect of genetically proxied inhibition of C3 on periodontitis risk (29). Both studies have agreed on the relevance of C3 inhibition in periodontitis. However, exploring C5 inhibition to change the risk of periodontitis has not been extensively studied, there is only robust biological plausibility for this hypothesis. Nevertheless, Eculizumab and Ravulizumab, both C5 inhibitors approved for the treatment of various medical conditions, have consistently demonstrated their efficacy and safety in human clinical trials (30). Their established safety record provides a compelling incentive for exploring their efficacy in treating other inflammatory diseases like periodontitis.

In our secondary analysis, we sought to explore the effect of C5 inhibition using an alternative way selecting IL-17, IL-1β, and TNF-lowering genetic variants in the C5 gene. We found supporting evidence consistent with the reduced risk obtained from our primary analysis; however, the results were not as robust as the primary causal estimate. In this analysis, we selected independent SNPs from the vicinity of the C5 gene locus associated with IL-17 plasma levels, and we observed a reduced risk of periodontitis aligning with our primary findings. On the other hand, we failed to find robust evidence when considering IL-1β, IL-6 and TNF as downstream biomarkers for C5 inhibition. In the mouse model study, IL-17, as well as IL-1β, IL-6 and TNF were inhibited after the administration of PMX-53 (13). The limited number of SNPs retained for our secondary analysis could explain why we failed to find compelling evidence.

Additionally, even though IL-17, IL-1β and TNF were proven to be relevant in mouse models, it is important to question their relevance in the C5-periodontitis axis among humans. Hence, this particular research field requires additional studies. While C5 inhibition directly altered these cytokines in mice, a transfer of the results to humans is uncertain as the reliability of mouse models for the investigation of human inflammatory diseases has been a concern so far. Gene expression profiling of mice and humans during endotoxemia suggested a relationship between the human genes and mouse orthologs and vice versa (31). Whether this can be extrapolated to include different inflammatory diseases like periodontitis is uncertain but worth investigating. Interestingly, the complement C3 inhibitor compstatin, renowned for its specificity for primate C3 was inactive against C3 from lower mammals (32). Testing potential therapies in compatible preclinical models remains essential to gain the closest insights into how this drug would function in humans. Our secondary analysis findings inspire further exploration of the biomarker panel. We aim to identify dependable biomarkers associated with C5 inhibition, pinpointing their proximity to C5 activation.

Our study’s notable strength is that we applied a novel IV approach to test whether a druggable protein can be a potential therapeutic target for periodontitis. Nevertheless, the study has limitations. First, the exposure and outcome association estimates were derived from individuals with European ancestry. Linkage disequilibrium patterns can differ between populations and may not extend to other ethnic groups, limiting our findings’ generalizability to other ethnicities (23). Although plausibly selected, we could not validate our IVs based on mRNA expression due to the unavailability of such data. Additionally, since we selected the SNPs from the vicinity of a single gene region, we were unable to apply pleiotropy-robust MR methods. Finally, when we considered IL-17, IL-1β, and TNF as downstream biomarkers, we obtained reduced SNP set for the genetic indexing of C5 inhibition. Future GWAS with larger sample size that identify additional SNPs associated with the phenotype have the potential to reassess our current findings, offering enhanced insights into the complex interplay between C5 inhibition and cytokine regulation.

Genetic studies of human populations are increasingly used as drug discovery and development research tools. These studies can facilitate the identification and validation of therapeutic targets. Our findings suggest prioritizing C5 inhibition as a potential treatment for periodontitis; nevertheless, the efficacy of C5 inhibition in periodontitis is yet to be assessed in preclinical and clinical trials. Further research in this direction could unveil novel therapeutic avenues akin to those explored for C3 intervention.

## Data Availability

The original contributions presented in the study are included in the article/**Supplementary Material**. Further inquiries can be directed to the corresponding author.
