# Supplementary material for: Using genetics to explore complement C5 as a druggable protein in periodontitis
Source: Front Immunol. 2024 Oct 8;15:1407431. doi: 10.3389/fimmu.2024.1407431 (PMC11493656; doi:10.3389/fimmu.2024.1407431)
Supplement: Supplementary file 1 [file DataSheet1.docx]

Supplementary Table 1. Associations of single nucleotide polymorphisms (SNPs) with plasma complement component 5 (C5) levels from the GWAS by Ferkingstad et al. (Ferkingstad et al., 2021) and periodontitis from the GWAS by Shungin et al. (Shungin et al., 2019)

|  |  |  |  | Estimates for C5 | | |  |  | Estimates for periodontitis | | |
| --- | --- | --- | --- | --- | --- | --- | --- | --- | --- | --- | --- |
| SNP | EA | OA | EAF | Beta | SE | P-value | F | R2 | Beta | SE | P-value |
| rs1035029 | A | G | 0.621 | 0.116 | 0.009 | 6.6e-42 | 184.0 | 5.2e-03 | 0.018 | 0.016 | 0.243 |
| rs10818445 | A | G | 0.380 | 0.045 | 0.009 | 1.7e-07 | 27.3 | 7.7e-04 | 0.028 | 0.016 | 0.093 |
| rs10984824 | T | C | 0.199 | 0.047 | 0.010 | 5.9e-06 | 20.5 | 5.8e-04 | 0.006 | 0.020 | 0.769 |
| rs10984989 | T | C | 0.035 | -0.143 | 0.022 | 1.2e-10 | 41.4 | 1.2e-03 | -0.071 | 0.064 | 0.270 |
| rs112913382 | G | A | 0.029 | -0.144 | 0.025 | 5.3e-09 | 34.1 | 9.7e-04 | -0.107 | 0.269 | 0.691 |
| rs117268816 | T | G | 0.032 | 0.136 | 0.024 | 1.3e-08 | 32.3 | 9.2e-04 | -0.037 | 0.063 | 0.557 |
| rs12002963 | T | C | 0.048 | -0.117 | 0.020 | 2.1e-09 | 35.8 | 1.0e-03 | -0.002 | 0.034 | 0.958 |
| rs12237868 | T | A | 0.091 | 0.136 | 0.014 | 4.0e-21 | 89.0 | 2.5e-03 | -0.021 | 0.026 | 0.418 |
| rs1359328 | C | T | 0.742 | 0.051 | 0.010 | 6.5e-08 | 29.2 | 8.3e-04 | 0.030 | 0.018 | 0.102 |
| rs141603224 | A | G | 0.004 | -0.369 | 0.066 | 2.4e-08 | 31.2 | 8.8e-04 | -0.009 | 0.085 | 0.913 |
| rs143936926 | C | G | 0.021 | -0.155 | 0.029 | 1.0e-07 | 28.3 | 8.0e-04 | 0.078 | 0.092 | 0.396 |
| rs148593837 | A | G | 0.014 | -0.189 | 0.035 | 8.0e-08 | 28.8 | 8.2e-04 | -0.962 | 0.521 | 0.065 |
| rs150631693 | G | A | 0.010 | -0.196 | 0.042 | 2.7e-06 | 22.0 | 6.2e-04 | 0.036 | 0.089 | 0.684 |
| rs2269063 | G | A | 0.025 | -0.134 | 0.026 | 2.7e-07 | 26.5 | 7.5e-04 | -0.014 | 0.049 | 0.768 |
| rs306784 | T | G | 0.309 | -0.063 | 0.009 | 1.7e-12 | 49.8 | 1.4e-03 | 0.004 | 0.016 | 0.786 |
| rs476951 | A | G | 0.116 | 0.067 | 0.013 | 2.6e-07 | 26.5 | 7.5e-04 | -0.012 | 0.025 | 0.635 |
| rs62572794 | T | C | 0.028 | -0.131 | 0.026 | 3.2e-07 | 26.1 | 7.4e-04 | -0.069 | 0.051 | 0.173 |
| rs62578445 | G | A | 0.052 | -0.184 | 0.019 | 4.9e-23 | 97.7 | 2.8e-03 | -0.034 | 0.038 | 0.371 |
| rs62580493 | T | C | 0.088 | -0.082 | 0.015 | 2.1e-08 | 31.4 | 8.9e-04 | -0.007 | 0.030 | 0.806 |
| rs62580495 | C | T | 0.008 | -0.232 | 0.046 | 4.8e-07 | 25.4 | 7.2e-04 | 0.001 | 0.158 | 0.996 |
| rs72760282 | C | T | 0.062 | -0.090 | 0.017 | 2.0e-07 | 27.0 | 7.7e-04 | -0.056 | 0.042 | 0.185 |
| rs76146514 | T | C | 0.051 | -0.164 | 0.018 | 6.3e-19 | 79.0 | 2.2e-03 | -0.042 | 0.042 | 0.316 |
| rs76960065 | A | C | 0.032 | -0.106 | 0.024 | 8.3e-06 | 19.9 | 5.6e-04 | -0.007 | 0.063 | 0.914 |
| rs77083332 | G | C | 0.043 | 0.146 | 0.021 | 1.5e-12 | 50.1 | 1.4e-03 | 0.002 | 0.043 | 0.969 |
| rs78152277 | C | T | 0.037 | -0.104 | 0.022 | 1.3e-06 | 23.4 | 6.6e-04 | -0.048 | 0.045 | 0.286 |
| rs79521092 | A | G | 0.019 | -0.173 | 0.031 | 2.6e-08 | 31.0 | 8.8e-04 | 0.315 | 0.414 | 0.446 |

EA, effect allele. OA, other allele. EAF, effect allele frequency. Beta, regression coefficient. SE, standard error. F, F statistics. R2, explained variability

Supplementary Table 2. Associations of single nucleotide polymorphisms (SNPs) with plasma interleukin 17 (IL-17) levels from the GWAS by Ferkingstad (Ferkingstad et al., 2021) and periodontitis from the GWAS by Shungin et al. (Shungin et al., 2019)

|  |  |  |  | Estimates for IL-17 A | | |  |  | Estimates for periodontitis | | |
| --- | --- | --- | --- | --- | --- | --- | --- | --- | --- | --- | --- |
| SNP | EA | OA | EAF | Beta | SE | P-value | F | R2 | Beta | SE | P-value |
| rs10818440 | T | C | 0.116 | 0.042 | 0.012 | 8.6e-04 | 11.1 | 3.2e-04 | 0.056 | 0.028 | 0.046 |
| rs118036389 | T | C | 0.067 | 0.053 | 0.016 | 8.5e-04 | 11.1 | 3.2e-04 | 0.084 | 0.057 | 0.138 |

EA, effect allele. OA, other allele. EAF, effect allele frequency. Beta, regression coefficient. SE, standard error. F, F statistics. R2, explained variability

Supplementary Table 3. Associations of single nucleotide polymorphisms (SNPs) with plasma interleukin 1β (IL-1β) levels from the GWAS by Ferkingstad et al. (Ferkingstad et al., 2021) and periodontitis from the GWAS by Shungin et al. (Shungin et al., 2019)

|  |  |  |  | Estimates for IL-1β | | |  |  | Estimates for periodontitis | | |
| --- | --- | --- | --- | --- | --- | --- | --- | --- | --- | --- | --- |
| SNP | EA | OA | EAF | Beta | SE | P-value | F | R2 | Beta | SE | P-value |
| rs117369936 | A | G | 0.041 | 0.074 | 0.021 | 4.1e-04 | 12.5 | 3.5e-04 | 0.124 | 0.114 | 0.277 |
| rs13284638 | T | C | 0.032 | 0.088 | 0.023 | 1.6e-04 | 14.2 | 4.0e-04 | 0.036 | 0.060 | 0.550 |

EA, effect allele. OA, other allele. EAF, effect allele frequency. Beta, regression coefficient. SE, standard error. F, F statistics. R2, explained variability

Supplementary Table 4. Associations of single nucleotide polymorphisms (SNPs) with plasma tumor necrosis factor (TNF) levels from the GWAS Ferkingstad et al. (Ferkingstad et al., 2021) and periodontitis from the GWAS by Shungin et al. (Shungin et al., 2019)

|  |  |  |  | Estimates for TNF | | |  |  | Estimates for periodontitis | | |
| --- | --- | --- | --- | --- | --- | --- | --- | --- | --- | --- | --- |
| SNP | EA | OA | EAF | Beta | SE | P-value | F | R2 | Beta | SE | P-value |
| rs75256583 | C | T | 0.983 | -0.096 | 0.03 | 1.4e-03 | 10.2 | 2.9e-04 | -0.089 | 0.098 | 0.365 |

EA, effect allele. OA, other allele. EAF, effect allele frequency. Beta, regression coefficient. SE, standard error. F, F statistics. R2, explained variability

Supplementary Figure 1: Leave-one-out IVW Analyses
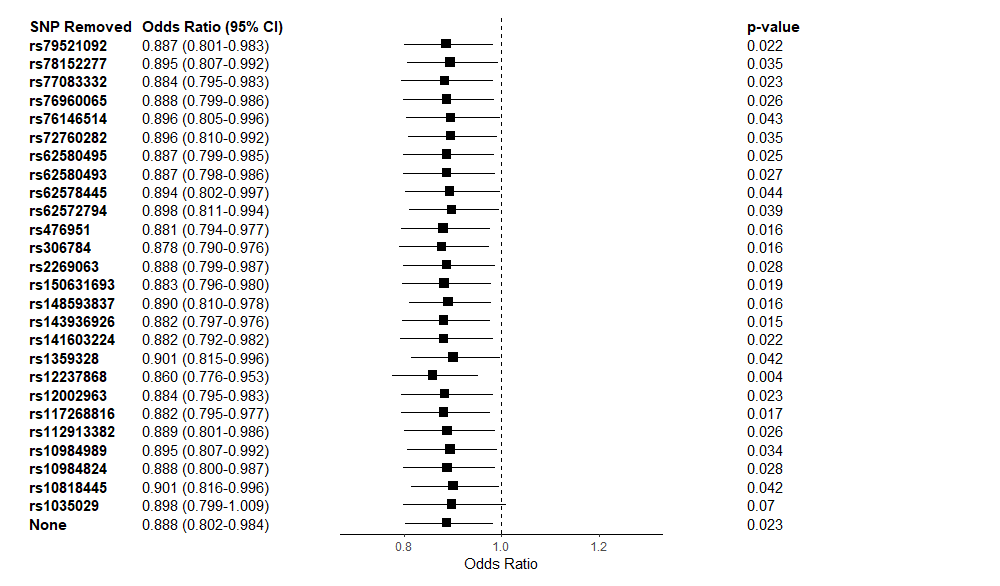


References

Ferkingstad, E., Sulem, P., Atlason, B. A., Sveinbjornsson, G., Magnusson, M. I., Styrmisdottir, E. L., Gunnarsdottir, K., Helgason, A., Oddsson, A., Halldorsson, B. V., Jensson, B. O., Zink, F., Halldorsson, G. H., Masson, G., Arnadottir, G. A., Katrinardottir, H., Juliusson, K., Magnusson, M. K., . . . Stefansson, K. (2021). Large-scale integration of the plasma proteome with genetics and disease. *Nature Genetics*, *53*(12), 1712–1721. https://doi.org/10.1038/s41588-021-00978-w

Shungin, D., Haworth, S., Divaris, K., Agler, C. S., Kamatani, Y., Keun Lee, M., Grinde, K., Hindy, G., Alaraudanjoki, V., Pesonen, P., Teumer, A., Holtfreter, B., Sakaue, S., Hirata, J., Yu, Y.‑H., Ridker, P. M., Giulianini, F., Chasman, D. I., . . . Johansson, I. (2019). Genome-wide analysis of dental caries and periodontitis combining clinical and self-reported data. *Nature Communications*, *10*(1), 2773. https://doi.org/10.1038/s41467-019-10630-1
